# Characteristics of New Peptides GQLGEHGGAGMG, GEHGGAGMGGGQFQPV, EQGFLPGPEESGR, RLARAGLAQ, YGNPVGGVGH, and GNPVGGVGHGTTGT as Inhibitors of Enzymes Involved in Metabolic Syndrome and Antimicrobial Potential

**DOI:** 10.3390/molecules25112492

**Published:** 2020-05-27

**Authors:** Urszula Złotek, Anna Jakubczyk, Kamila Rybczyńska-Tkaczyk, Paula Ćwiek, Barbara Baraniak, Sławomir Lewicki

**Affiliations:** 1 Department of Biochemistry and Food Chemistry, University of Life Sciences in Lublin, Skromna 8, 20-704 Lublin, Poland; urszula.zlotek@up.lublin.pl (U.Z.); paula.cwiek@onet.pl (P.Ć.); barbara.baraniak@up.lublin.pl (B.B.); 2 Department of Environmental Microbiology, University of Life Sciences in Lublin, St. Leszczyńskiego 7, 20-069 Lublin, Poland; 3 Department of Regenerative Medicine and Cell Biology, Military Institute of Hygiene and Epidemiology, Kozielska 4, 01-163 Warsaw, Poland; lewickis@gmail.com

**Keywords:** peptides, inhibitory, metabolic syndrome, cytotoxic properties, antimicrobial activity

## Abstract

The aim of this study was to determine the cytotoxic properties, influence on enzyme activity involved in metabolic syndrome, and antimicrobial activity of synthetic peptides with GQLGEHGGAGMG, GEHGGAGMGGGQFQPV, EQGFLPGPEESGR, RLARAGLAQ, YGNPVGGVGH, and GNPVGGVGHGTTGT sequences. Peptides have no cytotoxic effect on cells. The highest inhibitory effect on angiotensin converting enzyme I was noted for peptide GT-14 (IC_50_ = 525.63 µg/mL). None of the tested peptides had an influence on α-glucosidase. The highest α-amylase and lipase inhibitory activity was noted for GG-12 (IC_50_ = 56.72 and 60.62 µg/mL, respectively). The highest lipoxidase inhibitory activity was determined for peptide ER-13 (IC_50_ = 84.35 µg/mL). Peptide RQ-9 was characterized by the highest COX inhibitory activity (0.31 and 4.77 µg/mL for COX-1 and COX-2, respectively). Only peptide RQ-9 inhibited *S. enteritidis* ATCC 4931 growth (42–48%) in all tested concentrations (15.62–250 mg/mL).

## 1. Introduction

In recent years, proteins have been considered not only as a source of amino acids or building material for all body cells but also as a source of peptides that may have different biological properties. Peptides are delivered to the organism with protein food products. After hydrolysis in the gastrointestinal tract, they enter the bloodstream and are transported to the destinations of their action. Many them are antioxidants, inhibit the pathogenesis of metabolic syndrome, or have antimicrobial activity. Some peptides are isolated and identified from proteins, and others have been synthesized to study their properties. These compounds can be used as to reduce the development of diseases.

Metabolic syndrome (MS) is a risk factor of adverse pathophysiological conditions, including insulin resistance, disorders in sugar metabolism, visceral obesity, cardiovascular diseases, atherogenic dyslipidemia, and hypertension. The main role is played by such lifestyle factors as age, genetics, and socioeconomic status. Nowadays, there are growing numbers of data indicating that environmental risk factors, specifically air pollution, induce inflammatory processes that might play a key role in metabolic syndrome [1,2].

Diseases involved in MS are often associated with imbalance in the activity of enzymes that regulate the main processes in the organisms. Hypertension is the main chronic disease associated with malfunction of the most widely studied renin–angiotensin system. The angiotensin-converting enzyme (ACE) plays the most important role, as it catalyzes the formation of the potent vasoconstrictor angiotensin II (octapeptide) from inactive angiotensin I (decapeptide) and the breakdown of bradykinin, i.e., a vasodilator peptide. The excessive activity of ACE and production of angiotensin II cause a vasospasm and a direct increase in blood pressure, stimulation of cell proliferation, and excessive production of certain proteins. These changes lead to myocardial hypertrophy and damage as well as hypertrophy and stiffening of the vascular wall, which results in an increase in blood pressure and stimulation of the sympathetic nervous system. It causes blood vessels to contract, accelerates the heart rate, and increases the amount of blood ejected by the heart into vessels, which elevates pressure and, through long-term effects, causes damage to the heart and vessels. Furthermore, the secretion of the aldosterone hormone is elevated, which also increases blood pressure [3].

In recent years, great progress has been made in inhibition of the activity of the renin–angiotensin–aldosterone (RAA) system by using drugs that affect various links in the system. Inhibition of the activity of various types of cells involved in the RAA system is widely applied in the treatment of hypertension, cardiovascular diseases, and kidney diseases. Synthetic ACE inhibitors such as captroptril, enalapril, or ramipryl are commonly used as drugs in the treatment of hypertension, although they may cause serious side effects such as dry cough, dysgeusia, and angioedema [4]. Hence, it is necessary to search for new ACE inhibitors that may be obtained from food products and be safer. Such peptides have been identified from foods of various origins: plants [5], animals [6], or microorganisms. Their structure and activity differ, and the exact relationship between the structure and inhibition is still unknown. ACE inhibitory peptides represent di- or tripeptides such as the short chain peptides with sequences KVF, MKR, AKF, AMK, and GIL obtained from the enzymatic hydrolysates of lysozyme. In turn, there are several ACE inhibitory peptides with more than three amino acid residues in their structure: LIVGIIRCV from beef myofibrillar proteins [7] or peptides with sequences GHIITVAR, IGGIGTVPVGR, HIGNILSL, FMPGVPGPIQR, PNYHPSPR, AFPAGAAHW, HIITLGR, LAGNPAGR, MPGVPGPIQR, AGALGDSVTVTR, and INTLSGR obtained from sesame protein [8].

The other main risk factor of cardiovascular diseases is diabetes. There are several studies indicating a relationship between diabetes and an increased risk of hypertension [9]. Diabetes and carbohydrate disorders may be caused by the malfunction of enzymes involved in carbohydrate metabolism. One of them is α-amylase, whose activity is directly related to the development of type 2 diabetes, i.e., the most prevalent type of diabetes [10]. The enzyme is found in saliva and pancreas, and starch and glycogen are the substrate for its activity. Oligosaccharides released in the digestion process are further hydrolyzed into an absorbable monosaccharide: glucose. Glucose absorption into the bloodstream in patients with insulin disorders causes a sharp increase in the blood glucose level, which may lead to development of the disease [11]. Peptides with enzyme inhibitory activity involved in polysaccharide metabolism and exhibiting a potent anti-diabetic effect were described previously: CSSV, YSPR, SAAP, PGGP, and LGGGNT [12], or LPLLR [13]

Fats are energy-delivering molecules. Before being absorbed in the small intestine, they should be hydrolyzed by lipase into monoglycerol and free fatty acids. The main role in this process is played by pancreatic lipase, which can hydrolyze 50–70% of food-derived fat. The inhibition of excessive activity of this enzyme can be helpful in controlling postprandial hyperglycemia and/or reducing calorie intake, thereby bringing far-reaching health benefits in type 2 diabetes and obesity treatment [14]. One of the types of inhibitory compounds may be peptides whose activity depends on their structure and amino acid composition. There are a few peptide inhibitors, e.g., Ile-Trp-Ser and Tyr-Phe-Ser [15], CQPHPGQTC [16], or EITPEKNPQLR, and RKQEEDEDEEQQRE [17].

Obesity is regarded as systemic inflammation that is strongly influenced by the cyclooxygenase (COX) and lipoxygenase (LOX) pathways. Moreover, these inflammatory markers occupy an important position as they give insights into arising risk for cardiovascular disease (CVD), diabetes, and other metabolic disorders [18]. Therefore, the inhibition of inflammation enzymes may be effective in dietary therapy of obesity. In the literature, there are a few peptides with anti-inflammatory activity. Montoya-Rodríguez and Mejía reported that pure amaranth peptides HGSEPFGPR and RPRYPWRYT reduced the expression of LOX-1 and other factors involved in the inflammatory process, such as lipopolysaccharide (LPS)-induced inflammation or transforming growth factor-α (VCAM-1)-induced inflammation [19].

The main aim of our study was to estimate the role of synthetic peptides as inhibitors of selected enzymes associated with metabolic syndrome and determination of their antimicrobial activity and resistance to gastrointestinal enzymes. The peptides were described in our previous studies [20,21]. The research material was millet grains subjected to different temperature treatments (65 °C and 100 °C). Protein fractions from millet grains were isolated and hydrolyzed in gastrointestinal conditions. The hydrolyzates were a source of peptide fractions with molecular mass under 3.0 kDa with potent biopeptides.

## 2. Results

### 2.1. Cytotoxic Effect-Endothelial Cells

**Figure 1:**
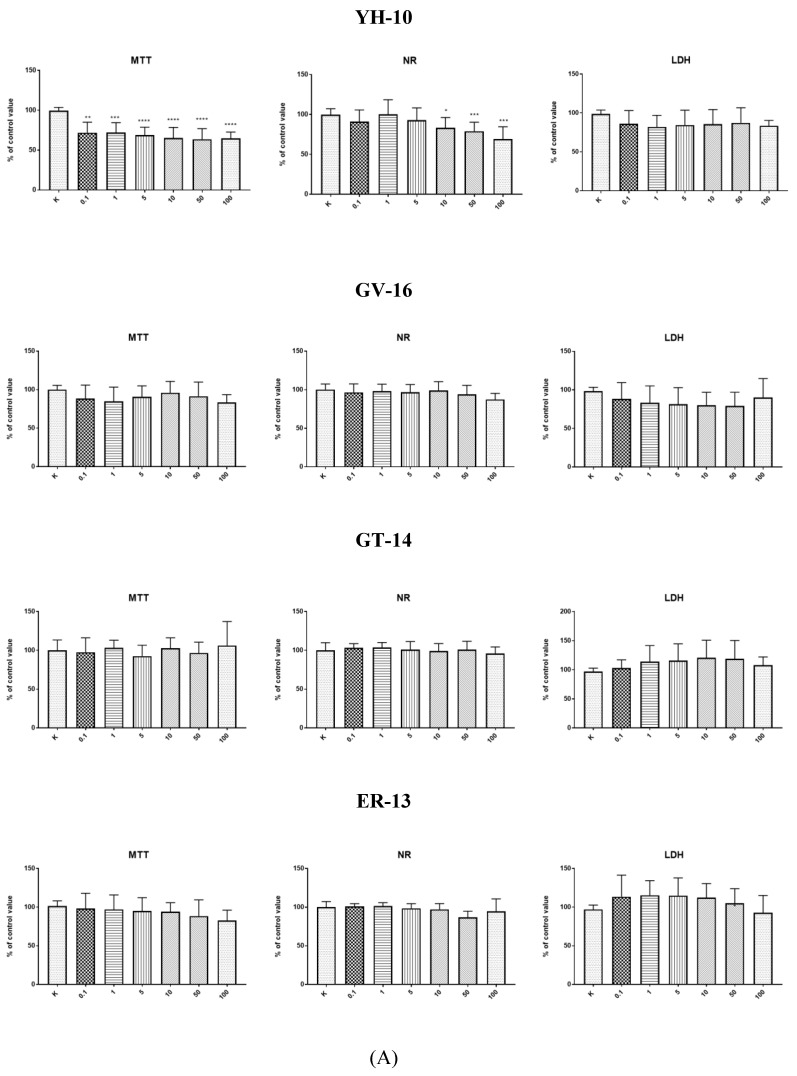

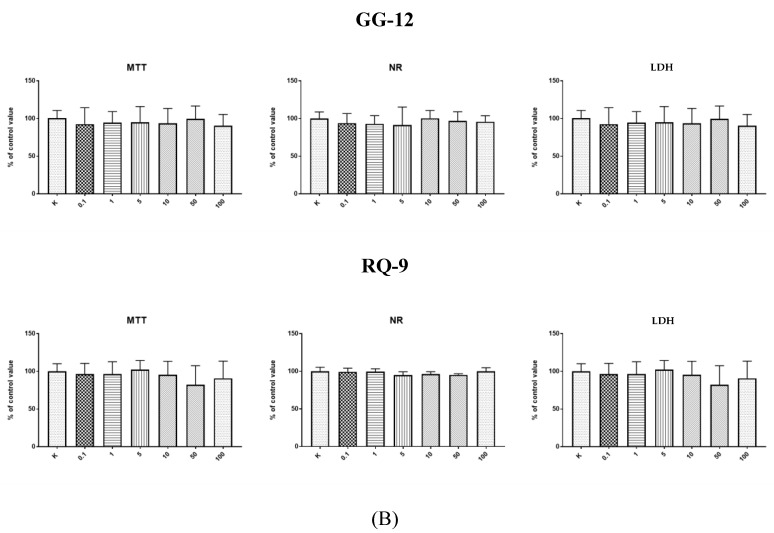
(**A**) Result of MTT, neutral red (NR), and LDH tests for peptides, * *p* < 0.05; ** *p* < 0.01; *** *p* < 0.001; **** *p* < 0.0001; (**B**) Result of MTT, neutral red (NR), and LDH tests for peptides.

It should be noted that bioactive peptides should have no cytotoxic effect on cells. 3-(4,5-dimethylthiazol-2-Yl)-2,5-diphenyltetrazolium bromide) (MTT), neutral red (NR), and lactate dehydrogenase (LDH) tests of all synthetic peptides were carried out. As shown by the data presented in Figure 1A,B, the HECa10 cell count declined statistically significantly after 24-h supplementation with peptide YH-10 at the concentrations of 10–100 µg/mL in both tests and in the full range of concentrations in the MTT test (MTT and NR test). There were no statistically significant differences in the LDH activity test. After 24-h incubation of endothelial cells (HECa10) with the other peptides at concentrations of 0.1–100 µg/mL, no statistically significant differences were observed in any of the tests (MTT, NR, LDH), compared to the values obtained for the control conditions.

### 2.2. Physicochemical Parameters

The physicochemical parameters of the peptides are shown in Table 1. The molecular mass of the tested peptides is between 955 and 1485 g/mol. Only one peptide (ER-13) has an instability index above 40, which may indicate that it is an unstable molecule. Almost all peptides were characterized by a positive grand average of hydropathicity (GRAVY) index characteristic for a hydrophobic compound, but only one peptide had a negative GRAVY index, indicating its hydrophilic nature.

### 2.3. Effect on Enzyme Activity

Table 2 shows the influence of synthetic peptides on enzymes involved in hypertension and disorders in the carbohydrate and lipid metabolism. Only peptide RQ-9 had no inhibitory effect on ACE activity. The highest IC_50_ = 525.63 µg/mL was determined for peptide GT-14. None of the tested peptides had an influence on α-glucosidase. However, the highest α-amylase and lipase inhibitory activity was noted for GG-12 (IC_50_ = 56.72 and 60.62 µg/mL, respectively). It should be noted that the IC_50_ values relative to α-amylase activity did not show statistically significant changes.

As shown in Table 3, the highest LOX inhibitory activity was determined for peptide ER-13 (IC_50_ = 84.35 µg/mL), and this value was statistically significantly different from the other results. In turn, peptide RQ-9 exhibited the highest COX inhibitory activity (0.31 and 4.77 µg/mL for COX-1 and COX-2, respectively). It should be highlighted that the lowest IC_50_ for COX was noted in the case of peptide GT-14 (IC_50_ = 4.43 µg/mL), but this value was not statistically significantly different from the result observed for peptide RQ-9. Table 4 and Table 5 show the kinetic parameters of all the tested enzymes with peptides.

### 2.4. Antibacterial Properties of Peptides

The antibacterial properties of the peptides were tested against bacteria *Escherichia coli* ATCC 25922, *Staphylococcus aureus* ATCC 29737, *Listeria monocytogenes* ATCC BBA-2660, *Bacillus cereus* ATCC 14579, and *Salmonella enteritidis* ATCC 4931 and yeast *Candida albicans* ATCC 90028. The results showed varied antimicrobial activity of the peptides. No antibacterial activity was observed against *B. cereus* ATCC 14579. The MIC values for the tested bacterial strain in the presence of the peptides were in the range of 62.50–250 mg/mL (Table 6).

**Figure 2:**
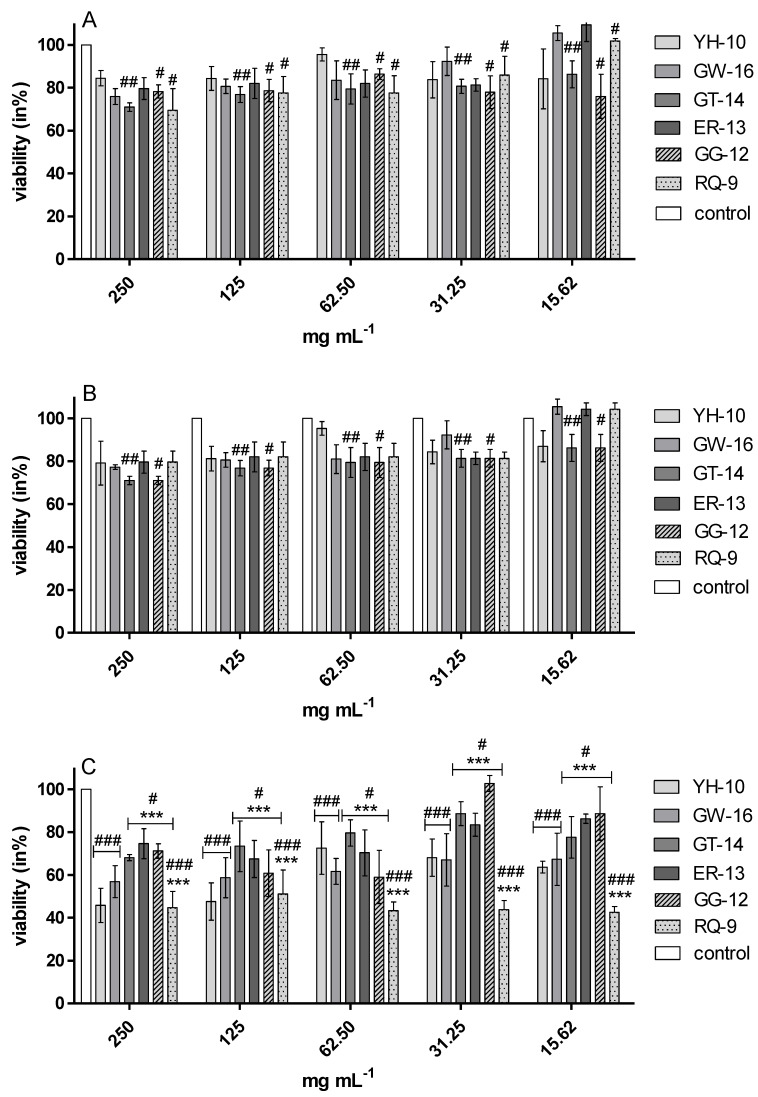
Viability (in %) of *E. coli* ATCC 25922 (**A**)*, S. aureus* ATCC 29737 (**B**), and *L. monocytogenes* ATCC BBA-2660 (**C**) in the presence of synthetic peptides samples (1–6), *** *p* < 0.001 versus the other samples, ###, ##, # *p* < 0.05 versus the control cultures of each bacteria.

**Figure 3:**
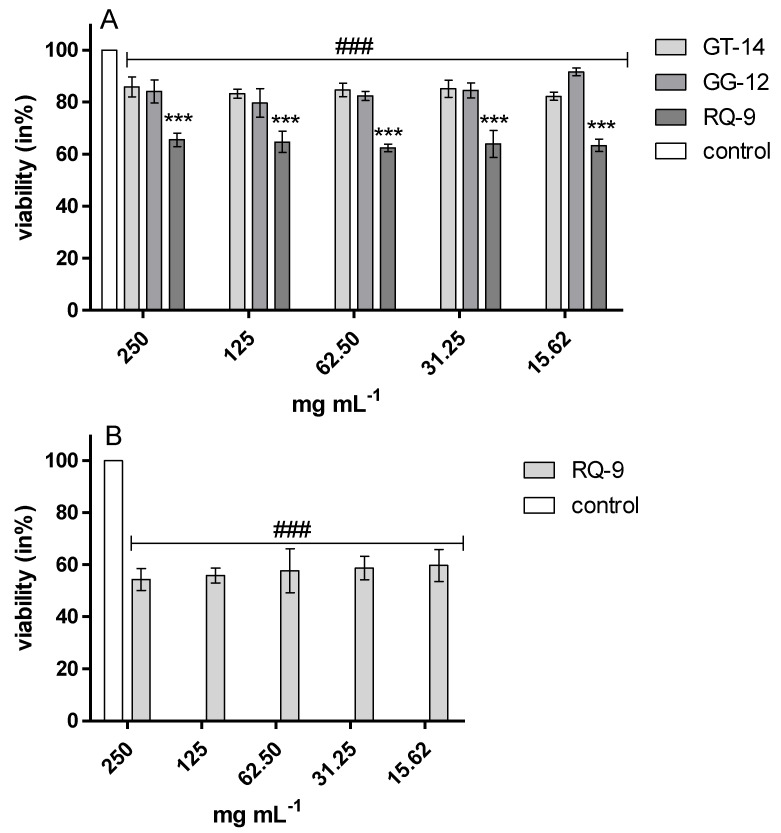
Viability (in %) *C. albicans* ATCC 90028 (**A**, samples GT-14, GG-12 and RQ-9) and *S. enteritidis* ATCC 4931 (**B**, sample RQ-9) in the presence of synthetic peptides samples, *** *p* < 0.001 versus the other samples, ### *p* < 0.05 versus the control cultures of yeast or bacteria.

These results were also confirmed by the resazurin reduction assay. It showed that the growth of *E. coli* ATCC 25922, *S. aureus* ATCC 29737, and *L. monocytogenes* ATCC 25922 in the presence of all the tested peptides was significantly inhibited versus control cultures (Figure 2). Peptide samples GT-14, GG-12, and RQ-9 induced the inhibition of *E. coli* ATCC 25922, *S. aureus* ATCC 29737, and *C. albicans* ATCC 90028 growth in the range of 30–40% and 15–60%, respectively (Figure 2A,B and Figure 3A). All samples inhibited the growth of *L. monocytogenes*, but RQ-9 inhibited bacterial growth the most potently (50–60%), even at the lowest concentration (Figure 2C). Peptide RQ-9 was different from the others, as it was the only one that inhibited *S. enteritidis* ATCC 4931 growth (42–48%) in all the tested concentrations (15.62–250 mg/mL) (Figure 3B).

## 3. Discussion

The search for natural substances in food with a potential therapeutic effect but no serious side effect has been carried out by researchers. Many studies have indicated that peptides exhibit bioactive properties, and food with high content thereof can be used to help treat metabolic syndrome diseases such as hypertension, insulin resistance, or obesity [22,23,24]. The application of natural peptides in foods to prevent the development of metabolic syndrome may be difficult, since any type of peptide used for food application must be safe for ingestion. Besides, the peptides should be stable and the method for derivation thereof must be effective and cheap. Designing novel synthetic peptides based on natural native peptides may be an alternative option for the application of peptides in foods.

In our study, all the synthetic peptides were tested to assess their cytotoxic effect. After 24-h incubation of endothelial cells (HECa10) with peptides GV-16, GT-14, ER-13, GG-12, and RQ-9 at different concentrations (0.1–100 µg/mL), no statistically significant differences were observed in any of the tests (MTT, NR, LDH) compared to the values obtained in the control conditions (Figure 1A,B). The results of incubation of the HECa10 cells at the concentrations of 10–100 µg/mL indicated significant differences in the MTT and NR tests. It should be noted that there were no statistically significant differences in the LDH activity test. This may suggest that the changes observed in the cells are not associated with the direct cytotoxic effect of YH-10, i.e., an increase in the LDH activity after cell membrane damage. They are rather associated with the modulation of cell metabolism by synthetic peptides. An additional argument for such a thesis is the concentration-independent effect of YH-10 on cell counts demonstrated in the MTT test. It is well known that some peptides affect cell metabolism. The effect of peptides depends on the cellular target (receptors, enzymes, inhibitors, etc.). Lorenzo et al. (2012) has shown in T-cells that altered peptide ligands (APL 1 and 2) affect the proliferation, cell cycle, and secretion of growth factors and cytokines, which in turn attenuate adjuvant arthritis (AA) and collagen-induced arthritis (CIA) in rat models [25]. In contrast, Conconi et al. reported inhibited cell adhesion, decreased cell proliferation, migration, and morphogenesis in Matrigel, and anti-angiogenic responses induced by small peptides carrying adhesion sequences (GRGDSP) (4)K [26]. In our research, new peptides were synthesized and therefore both cell effects and cell targets are unknown.

The HECa10 cells are murine high endothelial cells (HEC) from the peripheral lymph nodes, which were established as a non-transformed cell line, displaying and keeping the characteristic phenotype of the HEC of the tissue from where they are derived [27]. Besides their characteristic ability to recognize and select lymphocytes and specifically adhering cells, HECa10 produce characteristic blood vessel proteins, such as the angiotensin-converting enzyme, von Willebrand factor, VE-cadherin, and E-selectin [28]. Therefore, we used these cells as a model of the blood endothelial cell line. We believe that our synthetic peptides may pass through the digestive tract without any modification, and therefore may affect the metabolism of blood endothelial cell lines. We used murine endothelial cells, because we intended to use our synthetic peptides in mice with metabolic syndromes (induced or genetically changed) to find if there is any improvement of the health of endothelial cells.

The physicochemical parameters of the six synthesized peptides (YH-10, GV-16, GT-14, ER 13, GG-12, and RQ-9) were considered (Table 1). The peptides had molecular mass between 955.12 and 1485.59 g/moL, which corresponds 0.96 to 1.46 kDa. As reported by Wang and Li [29], this type of peptide may be transported via the paracellular route, which is the main intestinal transport pathway for high molecular mass peptides (500–1600 Da). To determine the peptide hydrophobicity, the grand average of hydropathicity (GRAVY) index defined by the sum of hydropathy values of all the amino acids divided by the peptide length was used. The peptides synthesized in this study had GRAVY indexes ranging from −1.315 to 0.011. A positive GRAVY index is obtained for a hydrophobic structure and a negative GRAVY index indicates hydrophilic compounds. The results indicate that the peptides have a globular (hydrophilic) rather than membranous (hydrophobic) structure [30,31]. The instability index provides an estimate of peptide stability during testing. A peptide with an instability index lower than 40 is expected to be stable. In turn, the value of >40 predicts that the peptide may be unstable [32]. Among tested peptides, only ER-13 was characterized by a higher value than 40, indicating its instability. Solubility in water was estimated based on the number of charged residues, isoelectric point (pI), and peptide length. As demonstrated by the data obtained in our study, only two peptides ER-13 and RQ-9 have good solubility in water. However, peptides characterized by poor solubility in water can have high biological activity as well. Although the solubility of peptides GKPVAVPA (GA-8) and GKHVAVHAR (GHA-8) described by Mirzaei et al. [32] was poor, these peptides exhibited ACE inhibitory activity.

In our study, we tested six synthesized peptides as compounds protecting against the development of metabolic syndrome. These disorders, i.e., glycemic index imbalance, hypertension leading to cardiovascular disease, dyslipidemia, and/or obesity, are an early factor of the potential future development of chronic conditions, such as type 2 diabetes [33]. Hypertension is regarded as one of the main causes of death worldwide and is associated with increased ischemic heart failure and coronary artery disease. One of the therapeutic methods for hypertension treatment is the use of ACE inhibitors, since reduction of the concentration of the most important vasoconstrictor (angiotensin II) by suppressing ACE is a key factor to achieve blood pressure balance and water balance in the body [34].

In our study, five tested peptides had an ACE inhibitory activity with the IC_50_ value in the range from 498.79 to 728.30 (µg/mL). It corresponds to a range of 0.43 to 0.52 mM. These values are higher than those obtained for peptides described in other studies, i.e., 2.5 µg/mL for peptide Thα1 [35] or 127 µg/mL for egg white protein (AFKDEDTEEVPFR) [36]. These differences may be associated with the different sequences and length of the peptides. Until recently, it was thought that only di- or tri-peptides with proline at the C-terminus have ACE inhibitory activity. There are several studies describing long-chain peptides without proline at the C-terminus, exhibiting the high potential of ACE inhibition [4,37]. Moreover, it has been observed that the presence of charged amino acids at the C-terminal of peptides plays the main role in the interaction with the ACE enzyme. The presence of hydrophobic amino acids at the C-terminal and aliphatic amino acids at the N-terminal position contributes to the ACE inhibitory action [38]. Several studies have also indicated that binding to ACE is influenced by hydrophobic amino acid residues (aromatic or branched chain) at three positions from the C-terminus of the peptide [39]. The amino acids such as izoleucine and valine in the aliphatic amino acid chain are described as factors increasing the inhibitory effect [40]. In turn, as indicated by Siow et al. [41], the combination of both hydrophilic and hydrophobic properties allows the peptide to act as an inhibitor. The authors suggest that a peptide with a “hydrophilic head” (consisting of Arg-His) and a hydrophobic tail (consisting of PAQPNYPWTAVLVF) would act in the same way. The K_m_ and V_max_ values at the different concentrations of the peptides were determined by the Lineweaver–Burk plot. The results showed a competitive inhibition mode of four purified peptides and an uncompetitive mode of one of them (Table 4). This result corresponds well with data reported by Villadóniga and Cantera [4], where the inhibition kinetic analysis showed that a peptide with the TTFHTSGY sequence was a fully functional ACE competitive inhibitor. It should be noted that ACE competitive inhibitory peptides are reported in literature most frequently [42]. A competitive inhibitor binds with free enzymes via interaction with an amino acid in the active site of the enzyme or with amino acids far from the active site. It should be noted that Captropril, i.e., the most common drug used in hypertension treatment, is also known as a competitive inhibitor of ACE [43].

Some of the factors that increase the risk of cardiovascular disease are overweight and obesity, which may also cause diabetes 2. The main cause of these health problems is the imbalance between the intake and conversion of calories into energy. To improve this situation, patients should incorporate physical activity it into their rhythm of the day and reduce the absorption of carbohydrates and lipids from their diet. The most important enzymes involved in carbohydrate hydrolysis and responsible for calorie absorption are α-amylase and α-glucosidase. The former hydrolyzes polysaccharides into oligosaccharides, whereas the latter catalyzes the final step to release absorbable monosaccharides [44]. The rapid increase in blood glucose can be reduced through the inhibition of enzymes involved in the release of glucose from foods, and this approach is employed in the management of type 2 diabetes [33]. According to the present data, all the tested peptides had α-amylase inhibitory activity but none of them inhibited α-glucosidase (Table 2). This may indicate that these peptides may not fully inhibit glucose absorption but may help reduce oligosaccharide secretion. Peptides from food inhibiting α-amylase and α-glucosidase were demonstrated in previous studies [13,45,46]. Moreover, the different amino acid compositions and sequences probably affect the bioactivity of peptides. As demonstrated by previous studies of the relationship between the structure and activity of peptides, the α-glucosidase and α-amylase inhibitory activities were strongly influenced by two hydrophobic amino acids, i.e., leucine and proline. It should be noted that the peptides analyzed in our study have a described amino acid sequence. Analysis of the relationship between the structure and activity also demonstrated that the C-terminal arginine has a positive effect on the polypeptide inhibitory activity [12], and this amino acid also is a component of the analyzed peptides. Almost all the peptides in this study are uncompetitive inhibitors (Table 4). These results correspond well with data described by Fu et al. [47], where longan pericarp proanthocyanidins were characterized as uncompetitive inhibitors of α-amylase activity. It should be noted that acarbose, a sugar analog inhibitor of α-amylase activity commonly used for diabetes treatment, shows competitive inhibition. Therefore, peptides or other food compounds with different structure may exhibit different inhibition behavior.

Another enzyme involved in the hydrolysis of high-energy food compounds is pancreatic lipase. This main lipolytic hydrolase in the small intestine in the digestive system is responsible for the hydrolysis of approximately 70% of dietary fats. Triacylogilcerols are digested by pancreatic lipase into monoacylglycerol and free fatty acids, which form mixed micelles in combination with bile acids, cholesterol, and lysophosphatidic acid [48]. An excessive amount of fatty acids flowing into cells may result in the formation of adipose tissue or the development of insulin resistance. Therefore, the inhibition of pancreatic lipase activity may prevent the occurrence of obesity as well and glucose metabolism disorders. Orlistat is one of the drugs used in obesity treatment whose action is based on the inhibition of pancreatic lipase activity. Although it shows satisfying effects on weight control, the serious side effects such as pancreatic damage, kidney disease, gastrointestinal disorders, and high cancer risk have limited the clinical application of this drug, especially for long-term treatment [49]. Therefore, new pancreatic lipase inhibitors in food compounds are being investigated. In our study, all the tested peptides exhibited pancreatic lipase inhibitory activity (Table 2) and were mostly characterized as uncompetitive inhibitors (Table 4). The activity of inhibitors depends on their structure and amino acid compositions. Peter and Bywater [50] suggest that the hydrophilic character is the main determinant of high inhibitory activity of peptides. This has explained the inhibitory activity of the tested peptides, which may be due to the varied amounts of hydrophilic residues (e.g., arginine, glutamic acid, glutamine, or threonine) in the peptide sequences.

Moreover, one of the factors of obesity and overweight is the inflammatory process in tissue. Lipid-mediated inflammation is strongly influenced by the cyclooxygenase (COX) and lipoxygenase (LOX) pathways, and these inflammatory markers play an important role as risk factors of diabetes and cardiovascular diseases [18].

In our study, all the examined peptides exerted a potential anti-inflammatory effect (Table 3), but ER-13 was characterized by the highest activity. It should be noted that the type of inhibition and potential anti-inflammatory activity are influenced by the structure and amino acid sequence of peptides. There a few reports about peptides with LOX and COX inhibitory activity, but the direct mechanism of this process has still not been elucidated. The results obtained in our previous study indicated that glycine-rich peptide fractions obtained from millet grains had potent anti-inflammatory activities [21]. As reported by Montoya-Rodríguez et al. [19], pure peptides described as HGSEPFGPR and RPRYPWRYT inhibited the expression of LOX-1 and other factors involved in the inflammatory process.

Although the peptides in this study are characterized by high inhibitory properties, there are a few strategies to improve their inhibitory effects. These include (1) the use of a mixture of peptides—there are literature reports on the synergistic effect of peptides [41,51,52], (2) effective transport of peptides, and (3) an effective method of encapsulation in the material from which the peptides will be released at the target site (for it may be used e.g., as edible films [53]).

Food-borne pathogens cause a great number of diseases with significant effects on human health. *E. coli, S. aureus, S. enteritidis*, and *L. monocytogenes* are responsible for food-borne infections [54]. *C. albicans* yeast is a commensal microorganism commonly colonizing the skin, gastrointestinal tract, genitourinary system, oropharynx, and upper respiratory tract without causing harm to healthy individuals [55,56]. However, in some cases, the yeast is associated with opportunistic infections in both animals and humans, especially in immunologically weak and immunocompromised patients such as those with HIV/AIDS [56,57]. In susceptible patients, *C. albicans* can enter the bloodstream by translocation across the mucosa of the gastrointestinal tract [55]. In recent years, more information about synthetic peptides is available, especially in relation to pathogenic microorganisms that cause e.g., harmful food-borne diseases in humans [56]. Antimicrobial peptides bind to bacterial cell membranes or mitochondrial membranes, which causes their disintegration and, consequently, cell death [58]. The antimicrobial activity of peptides is related not only to their physicochemical properties but the number and type of amino acids to [59]. Previously data indicated that a lack of Arg residue in the sequence of peptides is connected with less antibacterial ability [60]. It is in agreement with our data. On the other hand, Sousa et al. [61] reported that glycine/leucine-rich peptide inhibited the growth of Gram-negative bacteria such as *E. coli*. Our study indicated that the RQ peptide rich in Arg and Lys was characterized by higher antimicrobial activity compared with other tested peptides. Lum et al. [56] tested the anticandidal activity of synthetic peptides. Their results showed that hybrid peptides, KU2 and KU3, containing a mixed backbone of KABT-AMP and Uperin 3.6 had the most potent anticandidal activity against *C. albicans* 90028 with MIC values ranging from 16 to 128 mg/L. (= 0.016–0.128 mg/mL) [56]. Our results are in agreement with those reported in the previous study. We used resasurin assay based on the detection of metabolic activity of the cells. The redox dye resazurin (7-hydroxy-3H-phenoxazin-3-one 10-oxide) enters the cell in the oxidized form (blue) and is converted to the reduced form, resorufin (pink), mainly by enzymes in the electron transport system [62]. We estimated the anticandidal activity of synthetic peptide RQ-9, but with higher MIC values (15.62 mg/mL). Our study indicated antibacterial activity of RQ-9, GT-14, and GG-12 against *E. coli* ATCC 25922 (MIC = 250 mg/mL), YH-10, GV-16, GT-14, ER-13, GG-12 against Y, *S. aureus* ATCC 29737 (MIC = 250 mg/mL), and RG-9 against *L. monocytogenes* ATCC BAA-2660 (MIC = 15.62–250 mg/mL). The previous study indicated the antibacterial activity of peptide 35409 against *E. coli* ML 35 (43827) and *S. aureus* ATCC 29213 with MIC 22 and 350 µM, respectively. Cusimano et al. have demonstrated the antibacterial activity of synthetic peptides H2 and Tag against different strains of *L. monocytogenes* (MIC > 5 mg/mL) [63]. The growth of *S. enterica* was inhibited only by peptide RQ-9 (MIC = 15.62 mg/mL).

It should be emphasized that foods supplemented with the peptides described in this study are not only functional foods with health benefits but also sources of substances that inhibit the growth of pathogenic bacteria responsible for food poisoning. There are several studies of hydrolyzates or peptide fractions. In this study, the analysis was carried out only with peptides, as we do not have other compounds that could additionally affect enzyme activity.

## 4. Materials and Methods

The material of study was peptides: YGNPVGGVGH (YH-10), GEHGGAGMGGGQFQPV (GV-16), GNPVGGVGHGTTGT (GT-14), EQGFLPGPEESGR (ER-13), GQLGEHGGAGMG (GG-12), and RLARAGLAQ (RQ-9). The peptides were identified and described in our previous study [20]. The peptides were synthesized by Novazym Sc. (Poznań, Poland).

### 4.1. Analysis of the Physicochemical Characteristics of Synthetic Peptides

The physicochemical characteristics of the six novel synthetic peptides were predicted by online tools. The net charge and water solubility were estimated using the peptide property calculator available on https://pepcalc.com/. The ProtParamtool (http://web.expasy.org/protparam/) was used to analyze the isoelectric point (pI), instability index of peptides, hydrophobicity, and grand average of hydropathicity (GRAVY).

### 4.2. Determination of Cytotoxic Activity of Synthetic Peptides

The study was conducted according to the procedure described by Rokicki et al. [64]. HECa10 cells after 80–90% confluence were trypsinized (3 min, 37 °C), centrifuged (300 g, 5 min), counted, and plated in 96-well plates at a concentration of 1 × 10^4^ cells/well. Then, 24 h after seeding, cells were washed with phosphate-buffered saline (PBS) solution and culture medium (Dulbecco’s Modified Eagle Medium (DMEM) + 10% fetal bovine serum (FBS) + antibiotics, all from Life Technologies, Warsaw, Poland) was added with test factors in concentrations: 0.1; 1; 5; 10; 50; 100 µg/mL). Then, 24 h after addition of the compounds, proliferation tests (MTT, NR, LDH) were performed. The color intensity was determined using a Fluostar Omega microplate reader. Results are presented as the mean ± SD of 3 independent experiments (*n* = 12).

#### 4.2.1. MTT Test

Briefly, the cells were seeded in a 96-well culture plate at a concentration of 1 × 10^4^ cells/well. Then, 24 h after seeding, the cells were rinsed twice with PBS (Life Technologies, Warsaw, Poland) and resuspended in fresh growth medium. Peptide fractions were added at concentrations of 0 (control), 0.1, 1, 5, 10, 50, and 100 µg/mL. After 24 h incubation with the proteins, the assessment of cell metabolic activity based on MTT tests was performed (MTT kit, Sigma Aldrich, Poznań, Poland). The absorbance was measured at 570 nm with a FLUOstar Omega reader (BMG Labtech GmbH, Ortenberg, Germany). The results are presented as the percentage of the control values (mean ± SD). Each assay was performed in triplicate (*n* = 18).

#### 4.2.2. NR Test

The cells were seeded in a 96-well culture plate at a concentration of 1 × 10^4^ cells/well. Then, 24 h after seeding, the cells were rinsed twice with PBS (Life Technologies, Poland) and resuspended in fresh growth medium. Peptide fractions were added at concentrations of 0 (control), 0.1, 1, 5, 10, 50, and 100 µg/mL. After 24 h of incubation with the proteins, the uptake of the neutral red (NR kit, Sigma Aldrich, Poznań, Poland,) was assessed. The absorption was measured at a wavelength of 540 nm with the background cutoff at 690 nm (FLUOstar Omega, BMG Labtech GmbH, Ortenberg, Germany). The results are presented as the percentage of the control values (mean ± SD). Each assay was performed in triplicate (*n* = 18).

#### 4.2.3. LDH Test

The cells were seeded in a 96-well culture plate at a concentration of 1 × 10^4^ cells/well. Then, 24 h after seeding, the cells were rinsed twice with PBS (Life Technologies, Poland) and resuspended in fresh growth medium. Peptide fractions were added at concentrations of 0 (control), 0.1, 1, 5, 10, 50, and 100 µg/mL. After 24 h incubation with the proteins, activity of released from cells lactate dehydrogenase was assessed (Pierce Ldh Cytotoxicity Assay Kit, Life Technologies, Warsaw Poland).The absorption was measured at a wavelength of 540 nm with the background cutoff at 490 nm (FLUOstar Omega, BMG Labtech GmbH, Ortenberg, Germany). The results are presented as the percentage of the control values (mean ± SD). Each assay was performed in triplicate (*n* = 18).

### 4.3. Peptides as an Enzyme Inhibitory

For all inhibitory activity, IC_50_ was calculated. The IC_50_ value defined as the concentration of the extract inhibiting 50% of the enzyme activity was determined by measuring the enzyme inhibitory activity and peptide contents in each sample. The IC_50_ value was calculated from the plotted graph of the inhibition activity for the five different peptide concentrations.

#### 4.3.1. ACE Inhibitor Activity

The angiotensin converting enzyme was prepared according to the method described by Karaś et al. [20]. For ACE inhibitor activity, 5 μL of ACE was mixed with 5 μL of the samples and, after preincubation at 37 °C for 5 min, 5 μL of a 5 mM *N*-Hippuryl-l-histidyl-l-leucine (HHL) solution was added. The reaction was carried out at 37 °C for 60 min. Next, 0.1 M borate buffer with 0.2 M NaOH and 70 μL of an *o*-phthaldialdehyde OPA solution (20 mg/mL OPA in methanol) were added. The absorbance at 390 nm was measured using BioTek Microplate Readers (version, company, city, abbreviated state (if USA or Canada), country).

#### 4.3.2. α-amylase Inhibitory Activity

α-Amylase inhibitory activity (αAI) of the protein hydrolyzates and peptide fractions was measured according to the method described by Świeca, Baraniak, and Gawlik-Dziki [65]. α-amylase from hog pancreas (50 U/mg) was dissolved in the 100 mM phosphate buffer (containing 6 mM NaCl, pH 7.0). To measure the α-amylase inhibitory activity, a mixture of 25 μL of α-amylase solution and 25 μL of sample was first incubated at 40 °C for 5 min. Then, 50 μL of 1% (*w*/*v*) soluble starch (dissolved in 100 mM phosphate buffer containing 6 mM NaCl, pH 7) was added. After 10 min, the reaction was stopped by adding 100 μL of 3,5-dinitrosalicylic acid (DNS) and heating for 10 min. Then, the mixture was made up to 300 μL with double distilled water. After that, the absorbance at 540 nm was measured using BioTek Microplate Readers (Epoch™ 2 BioTek, Bad Friedrichshall, Germany). The final results were compared with the activity of the same amount of enzyme without the inhibitor. All assays were carried out in triplicate. For IC_50_ value determination, the inhibitory activity for four concentrations of samples was investigated.

#### 4.3.3. α-Glucosidase Inhibitory Activity

The α -Glucosidase Inhibitory Activity (αGIA) was measured with the method described by Jakubczyk, Świeca, Gawlik-Dziki, and Dziki [66]. Then, 10 μL of α-glucosidase (1 U/mL) and 20 μL 35 mmol/L *p*-nitrophenol were added to 0.5 mL of 0.1 mol/L phosphor buffer pH = 6.8. The reaction was incubated at 37 °C for 20 min. The α-glucosidase activity was observed as an increase in absorbance at 405 nm. For the αGIA measurement, 10 μL of α-glucosidase (1 U/mL) and 50 μL of the sample were added to 0.45 mL of 0.1 mol/L phosphor buffer pH = 6.8. After the incubation reaction at 37 °C for 5 min, 20 μL of 35 mmol/L *p*-nitrophenol was added. The reaction was incubated at 37 °C for 20 min. The α-glucosidase activity in the sample was expressed as an increase in absorbance at 405 nm.

#### 4.3.4. Pancreatic Lipase Inhibitory Activity

Lipase inhibitory activity was determined with the method described by Jakubczyk et al. [67]. The final volume of the reaction mixture was 150 μL (2 μL of the enzyme, 5 μL of the sample, 142 μL of 100 mM potassium phosphate buffer, pH 7.5, and 1 μL of a 100 mM *p*-nitrophenyl acetate (pNPA) solution in dimethyl sulfoxide (DMSO). Changes in absorbance at 234 nm were measured using BioTek Microplate Readers.

#### 4.3.5. Lipoxidase (LOX) Inhibitory Activity

The LOX inhibitory assay was carried out with the method described by Szymanowska et al. [68]. A solution of pure linoleic acid was used as a substrate. First, 157.2 µl of pure linoleic acid was mixed with 157.2 µL of Tween-20 and 10 mL of deionized water. The substrate was clarified with 1 mL of 1 M NaOH and M/15 phosphate buffer pH = 7.0 was added to the volume of 200 mL. The enzymatic reaction mixture contained 297 µL of M/15 phosphate buffer pH = 7, 2 µl of the substrate solution, and 1 µL of the enzyme solution. A mixture of 298 µl of phosphate buffer (pH 7.0) and 2 µL of the substrate solution was used to calibrate the spectrophotometer. Changes in absorbance at 234 nm were measured using BioTek Microplate Readers.

#### 4.3.6. COX-1 and COX-2 Inhibitory Activities

The effect of the peptide on COX-1 and COX-2 was measured using the COX Activity Assay kit from Cayman Chemical (Item No. 760151, Ann Arbor, MI, USA). COX activity was determined according to the instructions provided with the kit. The enzyme activity was assayed by calorimetric measurement of the appearance of oxidized *N*,*N*,*N*′,*N*′-Tetramethyl-*p*-phenylenediamine (TMPD) at 590 nm using BioTek Microplate Readers (Epoch™ 2 BioTek, Bad Friedrichshall, Germany).

### 4.4. Kinetics Parameters of Enzymes Inhibitors

Substrate solutions (5.0, 10.0, 15.0, and 20.0 mM) were prepared and used to determine the Michaelis constant (*Km*), the inhibition constant (*Ki*), and the maximum velocity (*V*max) of enzymes. The kinetic parameters were evaluated by Lineweaver–Burk’s method. The reaction conditions were the same as in the enzyme inhibitory activity assay.

### 4.5. Antimicrobial Activity

The synthetic peptides were tested against bacteria: *Escherichia coli* ATCC 25922, *Staphylococcus aureus* ATCC 29737, *Listeria monocytogenes* ATCC BBA-2660, *Bacillus cereus* ATCC 14579, and *Salmonella enteritidis* ATCC 4931 and yeast *Candida albicans* ATCC 90028. These strains were obtained from the American Type Culture Collection (ATCC, distributors: LGC Standards, Łomianki, Poland) and stored at 4 °C. All strains were cultured at 37 °C on Nutrient Broth (NB) medium.

#### 4.5.1. Determination of Minimum Inhibitory Concentration (MIC)

Serial twofold dilutions of peptides samples were made with Mueller Hinton Broth (MHB) and placed into 96-well plates to yield final concentrations ranging from 15.62 to 250 mg/mL. Then, 100 μL of bacterial or yeast culture were added. The bacterial and yeast suspension (100 µl) were prepared from an overnight culture and were adjusted to the inoculation of 10^8^ and 10^5^ CFU/mL, respectively. The wells with MHB medium or with microbial culture were the negative and positive control, respectively. The plates were incubated at 37 °C for 48 h. The minimal inhibitory concentration (MIC) is an indication of the lowest concentration of the tested extracts that prevents the visual growth of microorganisms.

#### 4.5.2. Estimation of Biotoxicity of Synthetic Peptides Using Resazurin Reduction Assay

Resazurine reduction assays were performed to estimate biotoxicity against the tested bacteria and yeast. This assay is based on the detection of the metabolic activity of the cells and measurement of the reduction capability of cells, which reflects the mitochondrial function and cell viability and shows time- and concentration-dependent microbial cell growth inhibition. The resazurin (7-hydroxy-3*H*-phenoxazin-3-one 10-oxide) enters the cell in the oxidized form (blue), where it is converted to a reduced form, resorufin (pink). After MIC estimation (4.6.1.), 20 μL of a 60-μM resazurin solution in PBS buffer were added to each well. After incubation (2 h, 37 °C), the viability of cells was monitored by measuring absorbance at 570 nm (reduced) and 600 nm (oxidized) [62] and calculating the bacteria viability (in %) against the control (bacteria growth without samples).

### 4.6. Statistical Analysis

All determinations were performed in triplicate. Statistical analysis was performed using STATISTICA 7.0 software for mean comparison using ANOVA with post-hoc Tukey Honestly Significant Difference Tukey′s (HSD) test at the significance level α = 0.05.

Data obtained from the experiment on cells were checked for normality of the distribution (Shapiro–Wilk test). The level of statistical significance was calculated: in the case of normal distribution—one-way ANOVA with Benferroni correction and student′s T test; in another case—non-parametric one-way ANOVA with correction Kruskal–Wallis and Mann–Whitney Test. The data were analyzed using the GraphPad Prism program (verse 5, GraphPad Software, Inc., La Jolla, CA, USA) at a significance level *p* < 0.05.

## Figures and Tables

**Table 1:** Physicochemical characteristic of peptides. GRAVY: grand average of hydropathicity.

Peptide Sequence (Abbreviation)	Molecular Weight g/mol	Net Charge	Theoretical pI	Instability Index	Aliphatic Index	GRAVY Index	Water Solubility
YGNPVGGVGH (YH-10)	956.03	+0.1	6.74	3.76 (stable)	58.00	−0.280	poor
GEHGGAGMGGGQFQPV (GV-16)	1485.59	−0.9	5.24	30.66 (stable)	24.38	−0.463	poor
GNPVGGVGHGTTGT (GT-14)	1210.27	+0.1	6.74	−13.99 (stable)	41.43	−0.314	poor
EQGFLPGPEESGR (ER-13)	1402.48	−2.0	4.25	92.12 (unstable)	30.00	−1.315	good
GQLGEHGGAGMG (GG-12)	1070.14	−0.9	5.24	−8.83 (stable)	40.83	−0.425	poor
RLARAGLAQ (RQ-9)	955.12	+2.0	12.00	8.89 (stable)	120.00	0.011	good

**Table 2:** IC_50_ value of enzymes involved in metabolic syndrome pathogenesis. ACE: angiotensin-converting enzyme.

Peptide (Abbreviation)	IC_50_ (µg/mL)			
	ACE	α-Amylase	α-Glucosidase	Lipase
YGNPVGGVGH (YH-10)	498.79 ± 3.66 ^a^	76.75 ± 7.9 ^a^	nd	102.25 ± 1.40 ^ab^
GEHGGAGMGGGQFQPV (GV-16)	728.30 ± 4.01 ^b^	66.22 ± 18.86 ^a^	nd	62.32 ± 4.44 ^ab^
GNPVGGVGHGTTGT (GT-14)	525.63 ± 3.08 ^c^	60.53 ± 2.35 ^a^	nd	104.21 ± 4.23 ^b^
EQGFLPGPEESGR (ER-13)	641.16 ± 2.18 ^d^	71.65 ± 10.94 ^a^	nd	76.81 ± 18.33 ^ab^
GQLGEHGGAGMG (GG-12)	561.60 ± 3.15 ^e^	56.72 ± 8.67 ^a^	nd	60.62 ± 17.20 ^a^
RLARAGLAQ (RQ-9)	nd	66.74 ± 5.91 ^a^	nd	97.31 ± 28.57 ^ab^

nd—not detected. All values are mean ± standard deviation for triplicate experiments. Different letters indicate significant differences (α = 0.05).

**Table 3:** IC_50_ value of enzymes involved inflammatory process pathogenesis.

Peptide (Abbreviation)	IC_50_ (µg/mL)		
	LOX	COX-1	COX-2
YGNPVGGVGH (YH-10)	121.66 ± 2.16 ^a^	16.61 ± 1.13 ^a^	15.53 ± 1.78 ^a^
GEHGGAGMGGGQFQPV (GV-16)	101.69 ± 2.72 ^b^	11.88 ± 0.79 ^b^	44.81 ± 2.03 ^b^
GNPVGGVGHGTTGT (GT-14)	185.71 ± 6.11 ^c^	14.84 ± 1.71 ^a^	4.43 ± 0.87 ^c^
EQGFLPGPEESGR (ER-13)	84.35 ± 4.62 ^d^	6.71 ± 0.47 ^c^	4.31 ± 0.99 ^c^
GQLGEHGGAGMG (GG-12)	140.26 ± 2.75 ^e^	7.61 ± 0.77 ^c^	16.85 ± 2.53 ^a^
RLARAGLAQ (RQ-9)	196.09 ± 3.43 ^c^	0.31 ± 0.01 ^d^	4.77 ± 1.01 ^c^

All values are mean ± standard deviation for triplicate experiments. Different letters indicate significant differences (α = 0.05).

**Table 4:** Kinetic parameters of enzymes involved in metabolic syndrome pathogenesis with peptides as inhibitor and type of inhibition.

	ACE						
	Reaction without Inhibitor	YH-10	GV-16	GT-14	ER-13	GG-12	RQ-9
K_m_ (mM)	0.56	1.29	3.29	2.99	1.86	2.21	na
V_max_	0.008	0.006	0.008	0.008	0.008	0.008	na
Type of Inhibition	-	uncompetitive	competitive	competitive	competitive	competitive	na
	α-amylase						
K_m_ (mM)	121.02	117.71	121.02	60.05	58.61	36.45	17.51
V_max_	1.04	0.86	0.73	0.42	0.56	0.45	0.30
Type of Inhibition	-	uncompetitive	noncompetitive	uncompetitive	uncompetitive	uncompetitive	uncompetitive
	Pancreatic Lipase						
K_m_ (mM)	306.40	306.40	94.92	100.77	306.40	145.30	47.33
V_max_	2.04	0.68	0.35	0.34	2.01	0.70	0.06
Type of Inhibition	-	noncompetitive	uncompetitive	uncompetitive	noncompetitive	uncompetitive	uncompetitive

**Table 5:** Kinetic parameters of enzymes involved in inflammatory process pathogenesis with peptides as inhibitor and type of inhibition.

	LOX						
	Reaction without Inhibitor	YH-10	GV-16	GT-14	ER-13	GG-12	RQ-9
K_m_ (mM)	1.41	1.41	1.41	1.41	1.41	4.16	5.08
V_max_	0.49	0.34	0.25	0.28	0.32	0.49	0.49
Type of Inhibition	-	noncompetitive	noncompetitive	noncompetitive	noncompetitive	competitive	competitive
	**COX-1**						
K_m_ (mM)	0.65	0.53	0.30	0.59	0.41	0.55	0.49
V_max_	0.42	0.32	0.23	0.34	0.27	0.32	0.31
Type of Inhibition	-	uncompetitive	uncompetitive	uncompetitive	uncompetitive	uncompetitive	uncompetitive
	**COX-2**						
K_m_ (mM)	0.12	0.07	0.09	0.11	0.12	0.07	0.08
V_max_	0.28	0.14	0.15	0.15	0.15	0.13	0.14
Type of Inhibition	-	uncompetitive	uncompetitive	uncompetitive	noncompeptitive	uncompetitive	uncompetitive

**Table 6:** Minimum inhibitory concentration (MIC) of tested peptides.

Peptide Sequence (Abbreviation)	*E. coli* ATCC 25922	*S. aureus* ATCC 29737	*S. enterica* ATCC 4931	*L. monocytogenes* ATCC BAA-2660	*C. albicans* ATCC 90028
MIC (mg/mL)					
YGNPVGGVGH (YH-10)	nd	nd	nd	125	nd
GEHGGAGMGGGQFQPV (GV-16)	nd	nd	nd	62.50	nd
GNPVGGVGHGTTGT (GT-14)	250	250	nd	250	nd
EQGFLPGPEESGR (ER-13)	nd	nd	nd	125	nd
GQLGEHGGAGMG (GG-12)	nd	250	nd	250	nd
RLARAGLAQ (RQ-9)	250	nd	15.62	15.62	15.62

nd—not detected.
